# Supplementary material for: Circulating miRNA Fingerprint and Endothelial Function in Myocardial Infarction: Comparison at Acute Event and One-Year Follow-Up
Source: Cells. 2022 Jun 2;11(11):1823. doi: 10.3390/cells11111823 (PMC9180782; doi:10.3390/cells11111823)
Supplement: Supplementary file 1 [file cells-11-01823-s001.zip › cells-1725662-supplementary materials/cells-1725662-Supplementary Figures.pdf]

SUPPLEMENTARY FIGURES

Circulating miRNA fingerprint and endothelial function in myocardial infarction: comparison at acute event and one-year follow-up

Ana Mompeón[1], Daniel Pérez-Cremades[1], Ana Belén Paes[1], Juan Sanchis[2], Luis Ortega-Paz[3,4], Rut Andrea[3,4], Salvatore Brugaletta[3,4], Manel Sabate[3,4], Susana Novella[1*], Ana Paula Dantas[3,4*†], Carlos Hermenegildo[1†]

[1] Department of Physiology, Faculty of Medicine and Dentistry, University of Valencia, Valencia, Spain. INCLIVA Biomedical Research Institute, Valencia, Spain.

[2] Cardiology Division, Hospital Clínico Universitario de Valencia (HCUV), INCLIVA Biomedical Research Institute, Universidad de Valencia, Centro de Investigación Biomédica en Red de Enfermedades Cardiovasculares (CIBERCV), Valencia, Spain.

[3] Institut d'Investigacions Biomèdiques August Pi i Sunyer (IDIBAPS), Barcelona, Spain.

[4] Institut Clinic Cardiovascular (ICCV), Hospital Clinic de Barcelona (HCB), Barcelona, Spain.

[†] These authors have contributed equally to this work

* Correspondence: susana.novella@uv.es (S.N.), adantas@clinic.cat (A.P.D)

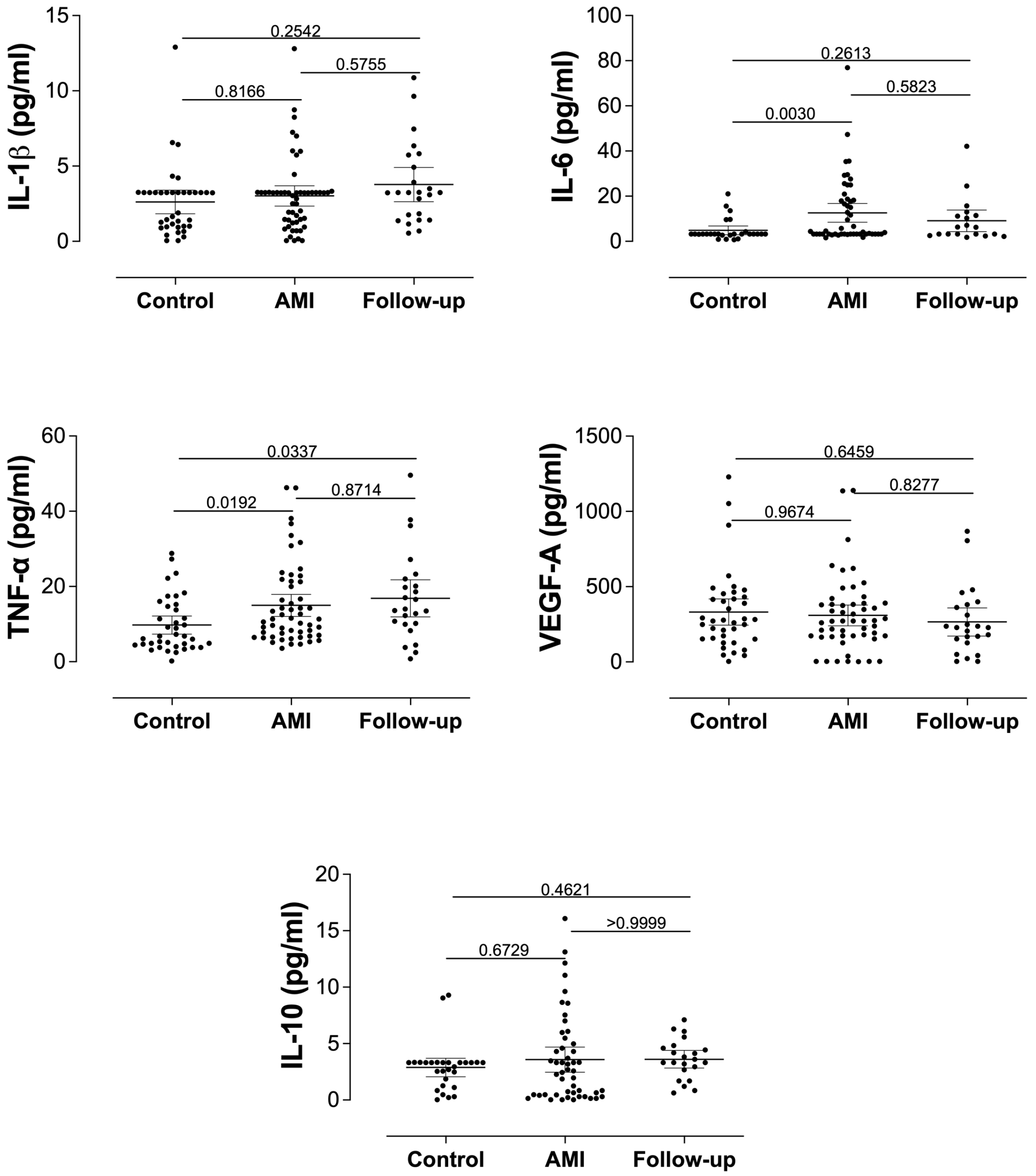

Figure S1. Serum levels of pro- and anti-inflammatory cytokines. Box and whiskers bars represent the mean ± SD of the concentration (pg/ml) of circulating levels of each cytokine detected in the serum of healthy volunteers (Control) and infarcted patients at the acute event (AMI) or after 1-year of follow-up (Follow-up). Shown are the pro-inflammatory cytokines interleukin 1-beta (IL-1β), interleukin 6 (IL-6), tumor necrosis factor (TNF-α) and vascular endothelial growth factor A (VEGFA); and the anti-inflammatory cytokine interleukin 10 (IL-10). P-values were calculated using Brown Forsythe and Welch ANOVA (Control n=51; NSTEMI n=53; Follow-up n=38).

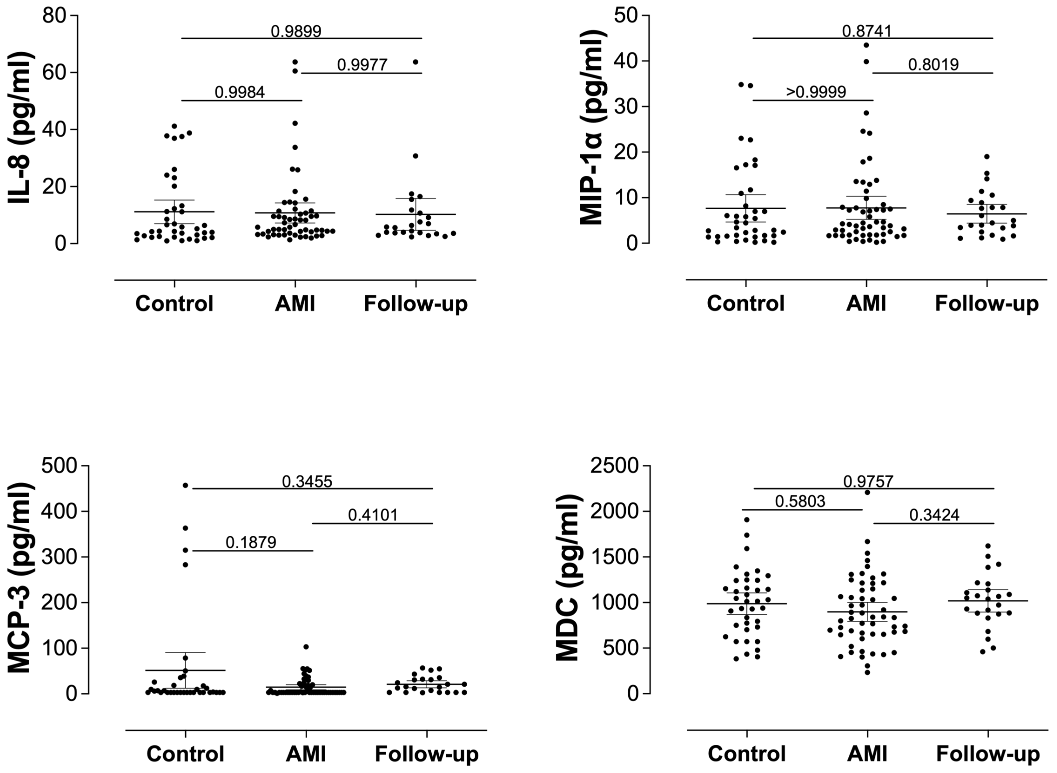

Figure S2. Serum levels of chemokines. Box and whiskers bars represent the mean ± SD of the concentration (pg/ml) of circulating levels of each chemokine detected in the serum of healthy volunteers (Control) and infarcted patients at the acute event (AMI) or after 1-year of follow-up (Follow-up). Shown are the chemokines interleukin 8 (IL-8), macrophage inflammatory protein 1-alpha (MIP-1α), monocyte chemotactic protein 3 (MCP-3) and macrophage-derived chemokine (MDC). P-values were calculated using Brown Forsythe and Welch ANOVA (Control n=51; NSTEMI n=53; Follow-up n=38).

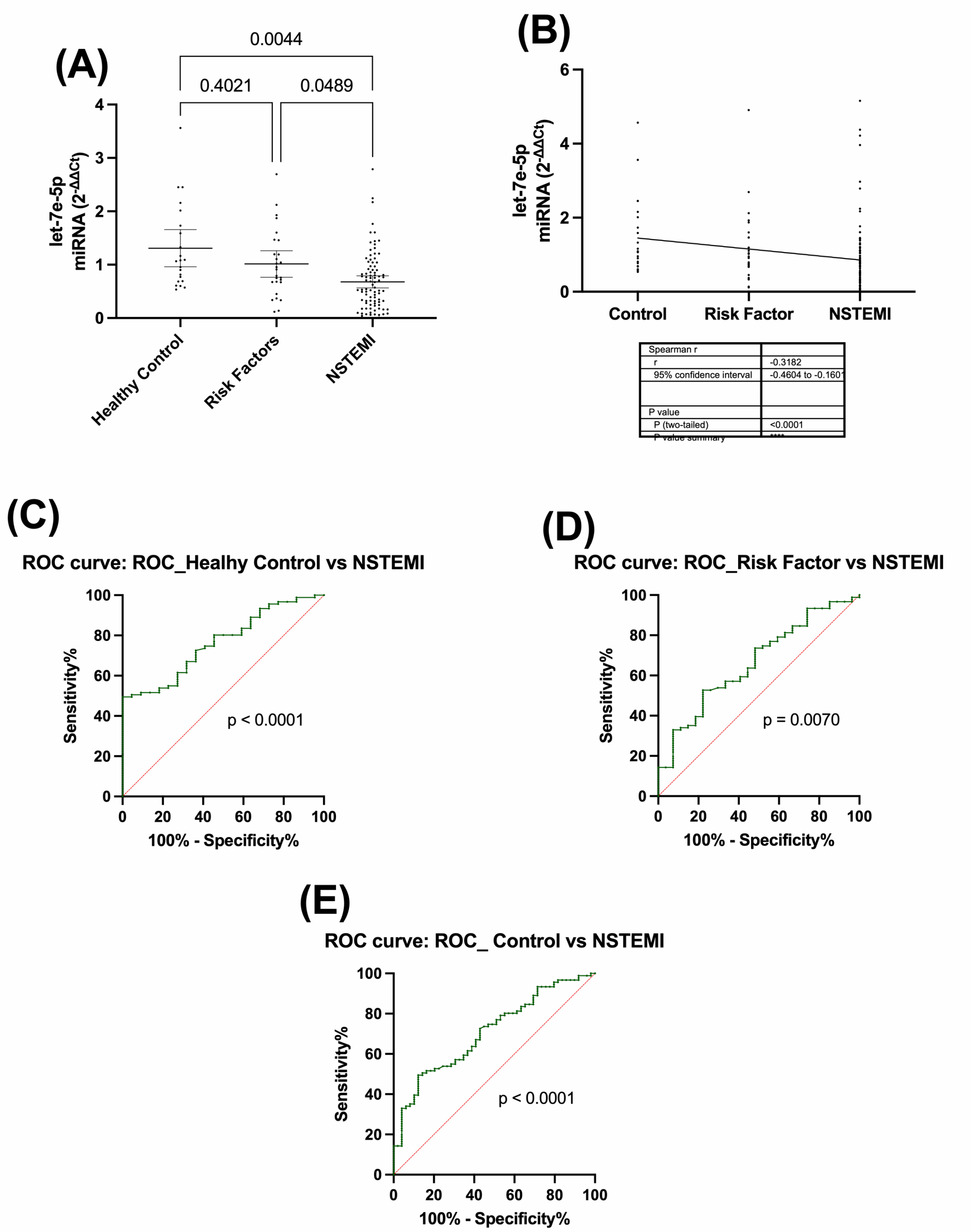

Figure S3. Sub-analysis of let-7e-5p expression by health status [healthy subjects, cardiovascular risk factors, and acute myocardial infarction (AMI)]. **(A)** Box and whiskers plot shows the differences of Means and SD of let-7e-5p expression analyzed using Brown Forsythe and Welch ANOVA. **(B)** show the correlation of let-7e-5p expression and health status calculated with Spearman r. ROC curves **(C – E)** show the performance of let-7e-5p measurements in the diagnosis of AMI differentiating it from healthy controls **(C)**, control with risk factors **(D),** and both controls combined **(E).** P-values were considered significant when <0.05.

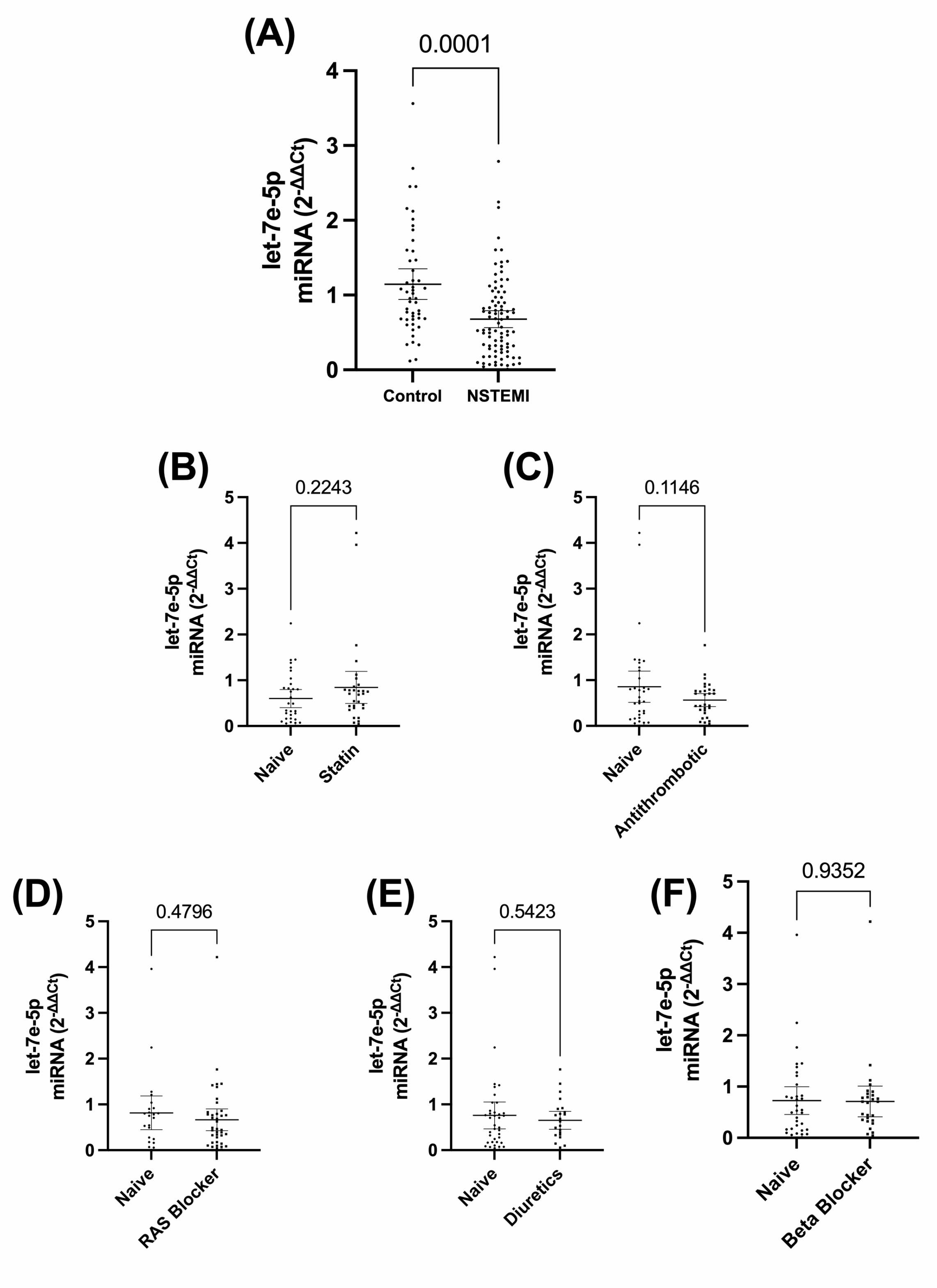

Figure S4. Sub-analysis of regulation of let-7e-5p expression by pharmacological treatments. Dot plots show the mean with 95% CI of let-7e-5p expression in control subjects and all patients in the acute event of myocardial infarction [AMI] **(A)**. AIM group was subdivided into patients with no previous exposure to a specific treatment (naïve groups), and those receiving statins **(B);** antithrombotic therapy **(C);** inhibitors of renin-angiotensin system [RAS] **(D);** diuretics **(E);** and beta-blockers **(F).** P-values were considered significant when <0.05.

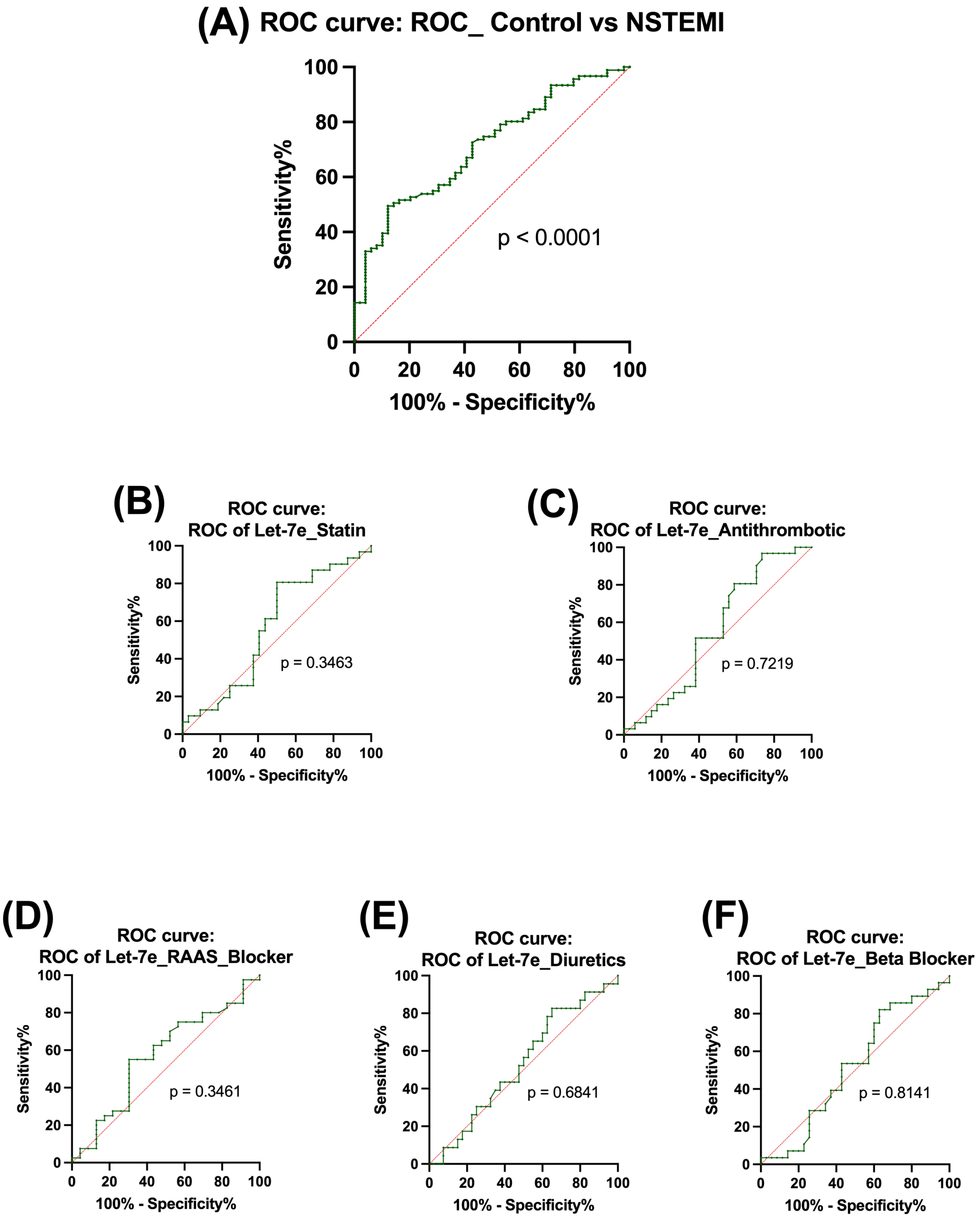

Figure S5. ROC curves show the performance of let-7e-5p measurements in the diagnosis of acute myocardial infarction [AMI] differentiating it from healthy controls **(A)**, as well as a sub-analysis of let-7e-5p expression performance by pharmacological treatments. Dot plots show the mean with 95% CI of let-7e-5p expression in control subjects and all patients in the acute event of myocardial infarction [AMI] **(A)**. AMI group was subdivided into patients with no previous exposure to a specific treatment (naïve groups), and those receiving statins **(B);** antithrombotic therapy **(C);** inhibitors of renin-angiotensin system [RAS] **(D);** diuretics **(E);** and beta-blockers **(F).** P-values were considered significant when <0.05.
